# Effects of Cannabidiol, ∆9-Tetrahydrocannabinol, and WIN 55-212-22 on the Viability of Canine and Human Non-Hodgkin Lymphoma Cell Lines

**DOI:** 10.3390/biom14040495

**Published:** 2024-04-19

**Authors:** Saba Omer, Suhrud Pathak, Mahmoud Mansour, Rishi Nadar, Dylan Bowen, Muralikrishnan Dhanasekaran, Satyanarayana R. Pondugula, Dawn Boothe

**Affiliations:** 1 Department of Anatomy, Physiology & Pharmacology, College of Veterinary Medicine, Auburn University, Auburn, AL 36849, USA; saba.scd@stmu.edu.pk (S.O.); mansoma@auburn.edu (M.M.); srp0010@auburn.edu (S.R.P.); 2 Department of Drug Discovery and Development, Harrison School of Pharmacy, Auburn University, Auburn, AL 36849, USArmn0012@auburn.edu (R.N.); drb0031@auburn.edu (D.B.); dhanamu@auburn.edu (M.D.)

**Keywords:** anti-lymphoma, apoptosis, exogenous cannabinoids, inflammation, mitochondria, non-Hodgkin’s lymphoma, oxidative stress

## Abstract

In our previous study, we demonstrated the impact of overexpression of CB_1_ and CB_2_ cannabinoid receptors and the inhibitory effect of endocannabinoids (2-arachidonoylglycerol (2-AG) and Anandamide (AEA)) on canine (*Canis lupus familiaris)* and human (*Homo sapiens*) non-Hodgkin lymphoma (NHL) cell lines’ viability compared to cells treated with a vehicle. The purpose of this study was to demonstrate the anti-cancer effects of the phytocannabinoids, cannabidiol (CBD) and ∆9-tetrahydrocannabinol (THC), and the synthetic cannabinoid WIN 55-212-22 (WIN) in canine and human lymphoma cell lines and to compare their inhibitory effect to that of endocannabinoids. We used malignant canine B-cell lymphoma (BCL) (1771 and CLB-L1) and T-cell lymphoma (TCL) (CL-1) cell lines, and human BCL cell line (RAMOS). Our cell viability assay results demonstrated, compared to the controls, a biphasic effect (concentration range from 0.5 μM to 50 μM) with a significant reduction in cancer viability for both phytocannabinoids and the synthetic cannabinoid. However, the decrease in cell viability in the TCL CL-1 line was limited to CBD. The results of the biochemical analysis using the 1771 BCL cell line revealed a significant increase in markers of oxidative stress, inflammation, and apoptosis, and a decrease in markers of mitochondrial function in cells treated with the exogenous cannabinoids compared to the control. Based on the IC50 values, CBD was the most potent phytocannabinoid in reducing lymphoma cell viability in 1771, Ramos, and CL-1. Previously, we demonstrated the endocannabinoid AEA to be more potent than 2-AG. Our study suggests that future studies should use CBD and AEA for further cannabinoid testing as they might reduce tumor burden in malignant NHL of canines and humans.

## 1. Introduction

Non-Hodgkin’s lymphoma (NHL) is the fifth leading cause of human cancer death and is the second fastest-growing cancer concerning mortality in people [1]. Likewise, lymphoma is one of the most common neoplasms afflicting dogs. It accounts for about 20% of all canine cancers and about 85% of blood cancers, with an incidence rate of 20–100 cases per 100,000 dogs and, in many respects, canine lymphoma is comparable to NHL in humans [2].

Both canine and human lymphomas exhibit a high rate of initial remission following conventional CHOP-based chemotherapies (cyclophosphamide, doxorubicin, vincristine, and prednisone). Nevertheless, 95% of dogs and 30% of humans will succumb to drug-resistant relapse [3,4,5].

To date, lymphoma remains a challenging disease condition, necessitating the need for novel strategies to improve the outcome of patients suffering from aggressive and/or therapy-resistant lymphoma.

Since the early seventies, cannabinoids have been used in human patients in cancer therapy due to their palliative effects (e.g., inhibition of chemotherapy-induced nausea, vomiting, appetite stimulation, and pain). In addition to their use in palliative interventions, cannabinoids have recently drawn renewed attention because of their diverse pharmacologic activities against cancer cells. These include cell growth inhibition, anti-inflammatory effects, and tumor regression effects in various xenograft animal cancer models [6,7,8].

Studies have shown that cannabinoids induce apoptosis in glioblastomas [9], PC-12 pheochromocytoma [10], CHP 100 neuroblastoma [11], breast [12], lung [13], prostate [14], and colon cancers [15], both in vitro and in vivo. In most cases, these diversified effects of cannabinoids were attributed to their ability to activate specific G protein-coupled receptors—CB_1_ and CB_2_. Within the body, a family of in vivo endogenously produced ligands normally bind these cannabinoid receptors, including the endocannabinoids Anandamide (AEA) and 2 acyl-glycerol (2AG). These endocannabinoids also bind the transient receptor potential (TRP) vanilloid type 1 (TRPV1) (as in the case of Anandamide), orphan G protein-coupled receptor (GPR-55), and peroxisome proliferator-activated receptors (PPARs) [16,17,18,19]. A study showed that CBD endocannabinoid decreases breast cancer aggressiveness by downregulating the helix–loop–helix protein Id-1, an inhibitor of basic helix–loop–helix transcription factors that promote human breast cancer progression [20].

In our previous study [21], we found a higher expression level of CB_1_ and CB_2_ receptors in canine and human B-cell lymphoma compared to T-cell lymphoma cell lines. We also described the cell growth inhibition and apoptotic effect of endocannabinoids on each of the canine and human NHL cell lines studied [21].

In the present study, we investigated the inhibition of lymphoma cell growth with selected exogenous phytocannabinoids (CBD and THC) and a synthetic cannabinoid (WIN). We also explored some of the toxicity mechanisms associated with treating canine and human lymphoma cells with selected phytocannabinoids and a synthetic cannabinoid. We hypothesized that exogenous cannabinoids, like endocannabinoids [21], have inhibitory effects on lymphoma cells’ viability.

## 2. Materials and Methods

### 2.1. Chemicals and Reagents

Phytocannabinoids (CBD and THC) and synthetic cannabinoid WIN were purchased from Sigma Aldrich (St. Louis, MO, USA). Methylthiazolyldiphenyl–tetrazolium (MTT) bromide cell viability assay was purchased from the American Type Culture Collection (ATCC) (Manassas, VA, USA). Penicillin–streptomycin solution was purchased from Thermo Fisher Scientific (Waltham, MA, USA). RPMI 1640 cell culture medium, embryonic cell (ES) qualified fetal bovine serum (FBS), and L- L-glutamine solution were purchased from EMD Millipore (Burlington, MA, USA). Phosphate-buffered saline (PBS), dimethylsulfoxide (DMSO), nicotinamide adenine dinucleotide (NADH), 2′,7-dichlorofluorescindiacetate (DCF-DA), pyrogallol, hydrogen peroxide (H_2_O_2_), phosphoric acid, 1-methyl-4-phenylpyridinium (MPP+), O-phthalaldehyde (OPT), L-glutathione (reduced), trichloroacetic acid, thiobarbituric acid, and phenylmethanesulfonyl fluoride (PMSF) were purchased from Sigma Aldrich (St. Louis, MO, USA). Glutamate was purchased from Alfa Aesar (Haverhill, MA, USA). Thermo-Scientific Pierce 660 nm Protein Assay reagent kit for protein quantification was purchased from Pierce (Rockford, IL, USA). The caspase substrates, AC-DEVD-AMC (Caspase-3 substrate), Ac-VETD-AMC (Caspase-8 substrate), and Ac-LEHD-pNa (Caspase-9 substrate), were purchased from Sigma Aldrich (St. Louis, MO, USA).

### 2.2. Canine Peripheral Blood Mononuclear Cells

Canine peripheral blood mononuclear cells (PBMCs) were separated from blood cell concentrates of healthy canines’ blood by density centrifugation on Ficoll–Paque gradient [22]. PBMCs were generously shared by Dr. Bruce Smith’s Lab (Auburn University) for this study.

### 2.3. Lymphoma Cell Lines

Authenticated canine B-cell lymphoma (BCL) 1771 (RRID:CVCL_0B18) and CLBL-1 (RRID:CVCL_L322) and T-cell lymphoma (TCL) cell line CL-1 (RRID:CVCL_L321) were a kind gift from Dr. Steven Suter’s Lab (North Carolina State University). Human Burkitt’s lymphoma cell line Ramos (RRID:CVCL_0597) was generously shared by Dr. Bruce Smith’s Lab (Auburn University, Scott-Ritchey Research Center).

### 2.4. Cell Culture

All cells were cultured in RPMI 1640 medium, supplemented with fetal bovine serum (10%), penicillin–streptomycin solution (1%), and L-Glutamine (1%), as we showed previously in our lab [21]. For the cell viability assay, all canine and human lymphoma suspension cells were grown and harvested via centrifugation. Cells were then seeded into 96-well plates at a density of 1 × 10^4^ cells/well. Lymphoma cells were incubated under standard conditions at 37 °C and supplemented with 5% CO_2_.

### 2.5. Treatment Design for Evaluating Lymphoma Cell Viability and Mechanisms of Cannabinoid-Induced Cytotoxicity

All drugs were dissolved in their recommended vehicles. CBD and THC were dissolved in ethanol, and WIN in DMSO. To determine the dose–cytotoxicity relationships, different concentrations of drugs were obtained by diluting each cannabinoid in a serum-enriched, fresh RPMI culture medium. For the control, cells were treated with the vehicle (Ethanol/DMSO) only. Cells were exposed to each drug in the range of 0.5–50 μM concentrations for 24 and 48 h. For biochemical analysis of drugs, canine 1771 lymphoma cells were typically exposed to 0 (vehicle only), 1 μM, and 50 μM concentrations of CBD, THC, and WIN individually for 24 and 48 h. The lowest (1 μM) and highest (50 μM) final concentrations were used to analyze the change in biochemical markers of oxidative stress, apoptosis, inflammation, and mitochondrial function with increasing doses and compared to the control untreated group. We used the cells treated with the vehicle as the control.

### 2.6. Evaluation of Cell Viability

The effect of cannabinoids on the viability of lymphoma cells was determined by 3-[4,5-dimethylthiazol-2-yl]-2,5-diphenyl tetrazolium bromide (MTT) assay. MTT assay measures the metabolic activity of cells, and there is a strong connection between metabolism and cell proliferation. The cells were plated in 96-well culture plates at 1 × 10^4^ cells per well in 100 μL of complete culture medium containing a range of concentrations from 0.5 to 50.0 μM of cannabinoids for 24 and 48 h at 37 °C in a humidified incubator. After incubation for specified times at 37 °C, MTT reagent (10 μL) was added to each well and incubated for 4 h, followed by the addition of 100 μL of solubilizing solution in each well to dissolve formazan crystals. Absorbance was recorded on a microplate reader at 570 nm wavelength. The effect of cannabinoids on cell viability was assessed as the percentage reduction compared to the viability of vehicle-treated cells, which was set as 100%.

### 2.7. IC50 Calculation

To calculate the IC50, a series of dose–response data was generated by treating cells with concentrations from 0 to 50 µM (5–8 concentrations). These concentrations were selected based on previous publications [23,24]. The experiment was repeated three times. Replica for each treatment = 12. Subsequently, linear regression analysis was performed using the dose–response curve fit in Prism. The concentration was transformed to log 10 and the data were normalized [25].

### 2.8. Total Protein Quantification

Protein was quantified using Pierce™ BCA Protein Assay Kit (Catalog number: 22662) according to the manufacturer’s instructions (Thermo Fisher Scientific). Multiple standard concentrations of bovine serum albumin (BSA) were used for the construction of a standard curve and extrapolation of protein concentration in each sample.

### 2.9. Biochemical Analysis

#### 2.9.1. Hydrogen Peroxide (H_2_O_2_) Content

The content of hydrogen peroxide (H_2_O_2_) in the control and treated 1771 canine B-cell lymphoma cells was measured using the Abcam Hydrogen Peroxide Assay kit (ab138874). A standard curve for H_2_O_2_ was obtained using a fluorometric plate reader (BioTek Synergy HT plate reader, BioTek, VT, USA) at 335 nm (excitation wavelength) and 390 nm (emission wavelength). The H_2_O_2_ content in the vehicle-treated negative control and cannabinoid-treated supernatant from each sample was calculated from the slope obtained from a standard curve. The results were expressed as H_2_O_2_ µmol/mg protein [26].

#### 2.9.2. Lipid Peroxidation

The content of lipid peroxide in the vehicle-treated control and cannabinoid-treated 1771 canine B-cell lymphoma cells was measured spectrophotometrically at 532 nm by measuring the malondialdehyde (MDA) content in the form of thiobarbituric acid-reactive substances (TBARS) using a plate reader (BioTek Synergy HT plate reader). The findings were expressed as µmol/mg protein [27,28,29].

#### 2.9.3. Reactive Oxygen Species (ROS) Generation

The generation of reactive oxygen species (ROS) in the control (vehicle-treated) and cannabinoid-treated 1771 canine B-cell lymphoma cells was estimated via spectrofluorometry by measuring the conversion of non-fluorescent chloromethyl-DCF-DA (2′,7-dichlorofluorescindiacetate, DCF-DA) to fluorescent DCF using an excitation wavelength of 492 nm and an emission wavelength of 527 nm. The results were reported as relative fluorescence intensity/mg protein [29,30,31].

#### 2.9.4. Nitrite Content

The nitrite content in the vehicle-treated control and cannabinoid-treated 1771 canine B-cell lymphoma cells was measured by a colorimetric method based on the Griess reaction. The final product of the nitric oxide (NO) oxidation pathway is nitrite (NO_2_^−^), which is measured as an expression of NO production. NO reacts with sulfanilamide under acidic conditions, leading to the production of diazonium ions. These diazonium ions are associated with N-(1-naphthyl) ethylenediamine to form chromophoric azo products, which can be measured spectrophotometrically at 545 nm, as described in [21,29,32].

#### 2.9.5. Glutathione (GSH) Content

The glutathione (GSH) content in the control and cannabinoid-treated 1771 canine B-cell lymphoma cells was measured via spectrofluorometry (327 nm excitation and 423 nm emission) using O-phthalaldehyde (OPT). The GSH measured was normalized to total protein content and reported as μmol relative GSH content/mg protein [29,33,34].

#### 2.9.6. NADH Content

A standard curve for NADH was obtained using a spectrophotometric plate reader (BioTek Synergy HT plate reader, BioTek, VT, USA), with measurements at 340 nm. The NADH content was calculated from the slope obtained from the standard curve and the results were expressed as µmol NADH/mg protein [35].

#### 2.9.7. Mitochondrial Complex-I Activity

NADH oxidation to NAD+ is catalyzed by mitochondrial complex-I (NADH dehydrogenase). The cell homogenate obtained from the vehicle-treated control and cannabinoid-treated 1771 canine B-cell lymphoma cells was added to PBS, and conversion of NADH to NAD+ was measured spectrophotometrically at 340 nm [29,34,36].

#### 2.9.8. Interleukin Converting Enzyme-I (ICE-1) Activity

The ICE-1 (also known as caspase-1) activity was measured using spectrofluorometry with a plate reader (BioTek Synergy HT plate reader, BioTek, VT, USA). AC-YVAD-AMC (10 µM) was used as a substrate, and the product was measured at 360 nm/460 nm. The ICE-1 activity in the vehicle-treated control and cannabinoid-treated supernatants was expressed as relative fluorescence intensity (RFU)/mg protein [37,38].

#### 2.9.9. Cyclooxygenase (COX) Activity

Cyclooxygenase (COX) activity was quantified spectrophotometrically. N,N,N′,N′-Tetramethyl-p-phenylenediamine dihydrochloride (TMPD) substrate was used to measure the COX activity in the vehicle-treated control and cannabinoid-treated supernatants. The product formed from the cleavage of TMPD by COX was measured at 600 nm. The COX activity in the vehicle-treated control and cannabinoid-treated supernatant was expressed as the formation of product/mg protein [39].

#### 2.9.10. Caspase-3, -8, and -9 Activities

The spectrofluorimetric method employing a plate reader (BioTek Synergy HT plate reader, BioTek, VT, USA) was used to measure caspase activities. A total of 10 µM of non-fluorogenic substrate AC-DEVD-AMC (for caspase-3); Acetyl-Asp-Glu-Val-Asp-7-amido-4-methylcoumarin) and AC-IETD-AMC (for caspase-8); and Acetyl-Ile-Glu-Thr-Asp-7-Amino-4-methylcoumarin) and AC-LEHD_AMC (for caspase-9) was utilized to measure the corresponding caspase activities. The conversion of the non-fluorescent substrate to fluorescent AMC was quantified at 360 nm/460 nm (excitation/emission wavelength). The caspase-3, -8, and -9 activities in the vehicle-treated control and cannabinoid-treated supernatants were expressed as relative fluorescence intensity (RFU)/mg protein [38,40].

### 2.10. Statistical Analysis

For the statistical analysis of cell viability, biochemical data, and IC50 calculation, the range of the measured variables and their means, standard deviations (SDs), and standard errors (SE) were calculated using a statistical software (Prism 9, version 9.5.1, La Jolla, CA, USA). The data are presented as mean ± SD. The differences between the sample treatments were evaluated with Student’s *t*-test and non-parametric ANOVA, followed by Dunnett’s multiple comparison test; *p*-values less than 0.05 were considered statistically significant.

## 3. Results

### 3.1. Biphasic Effect of CBD, THC, and WIN on Canine and Human Non-Hodgkin Lymphoma (NHL) Cell Viability

The phytocannabinoids, cannabidiol (CBD) and ∆9-tetrahydrocannabinol (THC), used in this study are carboxylated forms of terpenophenolic compounds present in the cannabis plant. WIN, on the other hand, is a synthetic aminoalkylindole cannabinoid.

#### 3.1.1. Treatment with CBD and THC Phytocannabinoids Altered Cell Viability in Human and Canine Lymphoma Cells

**Figure 1:**
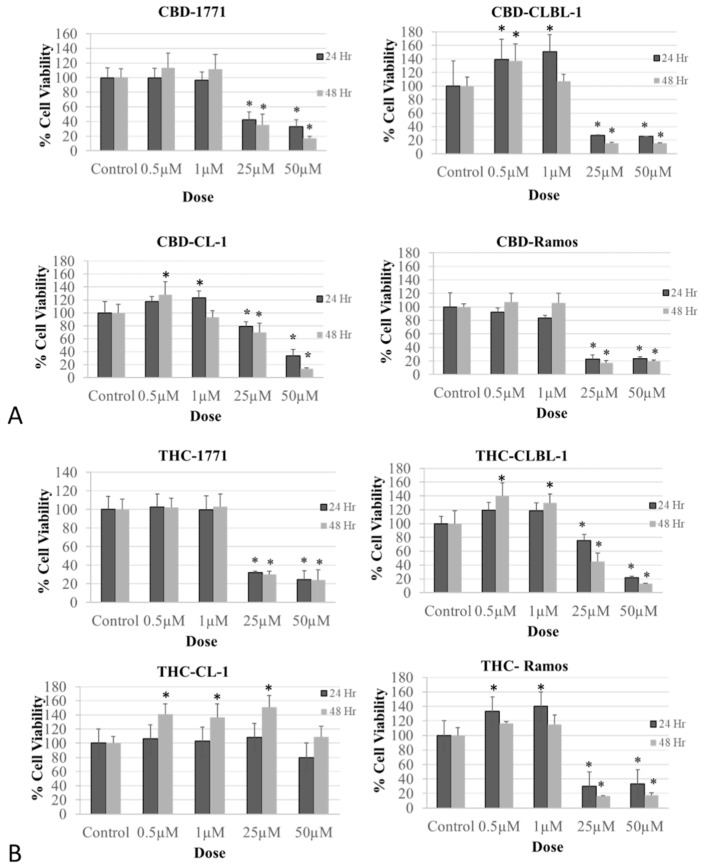
Biphasic effect of the phytocannabinoids cannabidiol (CBD) and ∆9-tetrahydrocannabinol (THC) on cell viability of canine and human non-Hodgkin lymphomas. (**A**). CBD treatment of B-cell lymphomas (canine 1771 and CLBL1, and human Ramos) and canine T-cell lymphoma (CL-1). (**B**). THC treatment of B-cell lymphomas (canine 1771 and CLBL1 and human Ramos). The experiment was repeated three times at 24 and 48 h. Replica for each treatment = 12. The results are expressed as (%) change compared to the 100% cell viability in vehicle-treated control. Values over 100% are due to increased cell growth. Data are depicted as mean ± SD. Data were evaluated with analysis of variance (ANOVA), followed by Dunnett’s multiple comparison test. Of note, no biphasic effect was detected in CL-1 canine TCL cells when treated with THC (**B**). Overall, CBD demonstrated a more potent inhibitory effect on cell viability against BCL than against TCL (**A**). (* statistically significant increase/decrease in cell viability compared to the control).

Treatment of canine B-cell lymphoma (1771 and CLBL-1) and human Ramos BCL cell lines with the phytocannabinoids CBD (Figure 1A) and THC (Figure 1B) demonstrated a biphasic effect with a stimulatory upshot at lower doses, followed by a significant reduction in cell viability at 25–50 μM concentrations (number of replicas for each treatment = 12, *p* < 0.0001). No such effect was detected in the canine CL-1 T-cell lymphoma cell line with THC treatment.

#### 3.1.2. Treatment with WIN Altered NHL Cell Viability in Human and Canine Lymphoma Cells

**Figure 2:**
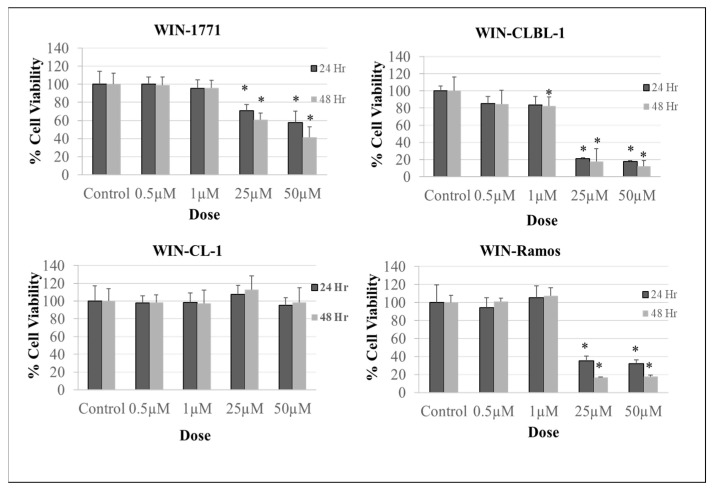
Effect of WIN on canine and human non-Hodgkin lymphoma cell viability. The WIN treatment decreased cell viability in canine and human Ramos B-cell lymphoma cell lines (1771, CLBL-1, and Ramos). No significant effects were detected in canine T-cell lymphoma cell line CL-1. The WIN treatment was at 0.5–50 µM concentrations for 24 h and 48 h. The experiment was repeated three times. Replica for each treatment = 12. The results are expressed as (%) change compared to the control, with mean ± SD. Data were evaluated using analysis of variance (ANOVA), followed by Dunnett’s multiple comparison test. (* statistically significant increase/decrease in cell viability compared to the control).

Like the effect of phytocannabinoids, the WIN treatment at 25–50 µM final concentrations significantly decreased cell viability (*p* < 0.0001) in both canine and human non-Hodgkin BCL when compared to the vehicle treatment (Figure 2). We could not demonstrate a significant inhibitory effect of WIN on CL-1 canine TCL cell viability.

#### 3.1.3. Treatment with CBD Does Not Affect Healthy Lymphocytes’ Viability

**Figure 3:**
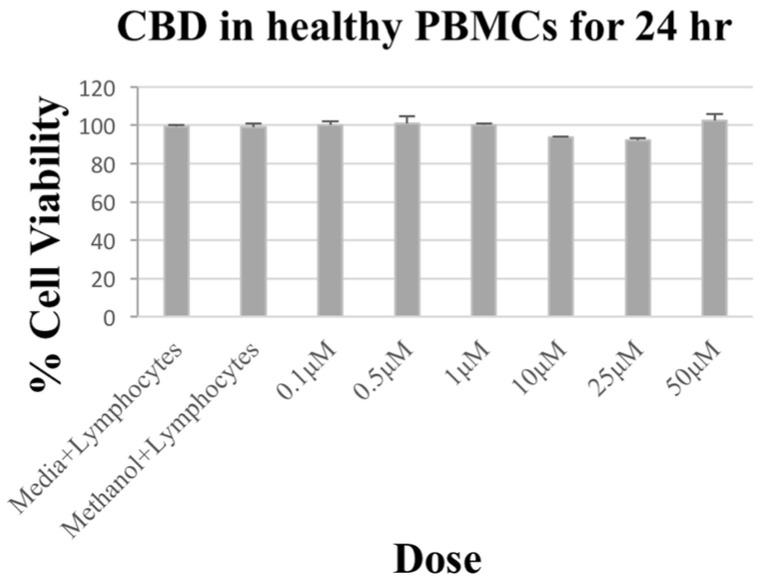
Effect of CBD on canine healthy lymphocytes’ viability. CBD treatment caused no significant effect in canine healthy lymphocytes. CBD treatment was at concentrations of 0.1–50 µM for 24 h. Results are expressed as (%) change compared to control, with mean ± SD.

Unlike lymphoma cells, treatment of healthy lymphocytes with CBD at concentrations from 0.1 to 50 µM for 24 h demonstrated no significant effect on these lymphocytes’ (PBMCs) viability (Figure 3).

### 3.2. IC50 Calculation of Phytocannabinoids and a Synthetic Cannabinoid Based on Canine and Human Non-Hodgkin Lymphoma (NHL) Cell Viability

To further study the inhibitory effects of phytocannabinoids (CBD and THC) and a synthetic cannabinoid (WIN) on NHL cell viability, we calculated the IC50 of each cannabinoid in each NHL cell line used in this study. IC50 is a parameter that estimates the concentration of a drug that results in 50% inhibition of cell concentration when treated.

The analysis of IC50 shows that the phytocannabinoid CBD is the most potent cannabinoid among the cannabinoids used in 1771, Ramos, and CL-1, and WIN was found to be the most potent cannabinoid in CLBL-1 cell line (Table 1 and Supplementary Materials). All the cannabinoids studied are more effective against the canine and human B-cell lymphoma cell lines than against the T-cell lymphoma CL-1 cell line. Among the B-cell lymphoma cell lines, all the cannabinoids studied are more effective against the CLBL-1 and Ramos B-cell lines than the 1771 B-cell line. CBD was found to be the only effective cannabinoid against CL-1 canine T-cell lymphoma cells in terms of IC50 when compared to the other cannabinoids used in the study (Table 1 and Supplementary Materials).

The maximum concentration used to generate the dose–response curve was 50 µM; for cell lines where IC50 could not be calculated within the treated concentrations, it suggests that the concentrations of the drug (0.1–50 µM) did not effectively inhibit cell viability.

### 3.3. CBD, THC, and WIN Treatments Induced Oxidative Stress in Canine 1771 B-Cell Lymphoma Cell Line

**Figure 4:**
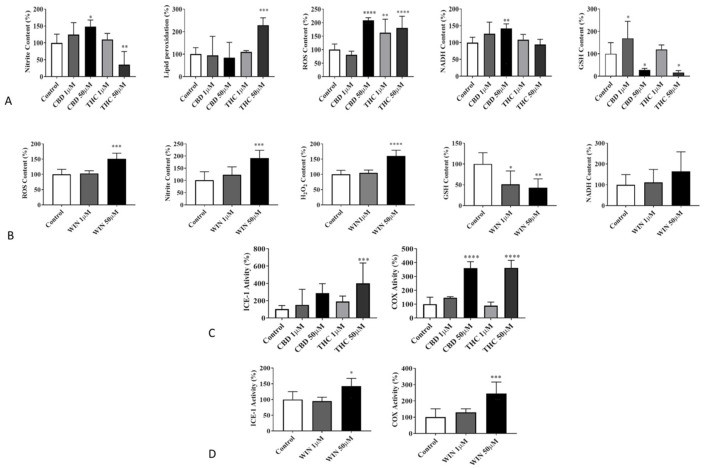
(**A**,**B**) Effects of CBD, THC, and WIN on markers of oxidative stress in 1771 canine lymphoma cells. All markers of oxidative stress were measured spectrofluorometrically. (**A**) CBD treatment is associated with a dose-dependent increase in nitrite content, reactive oxygen species (ROS) generation, and NADH and lipid peroxidation, and a simultaneous dose-dependent decrease in the GSH content in lymphoma-treated cells when compared to the control. THC demonstrates a dose-dependent increase in lipid peroxidation and ROS content and a decrease in GSH and nitrite contents. A significant effect could not be demonstrated in the cellular content of NADH in cells treated with THC. (**B**) The synthetic cannabinoid (WIN) shows a significant dose-dependent increase in nitrite, ROS, and H_2_O_2_ contents and a decrease in GSH compared to the vehicle-treated control cells. (**C**,**D**) Effects of exogenous cannabinoids on Interleukin-1β-converting enzyme (ICE-1) and cyclooxygenase (COX) activities, showing a significant increase in lymphoma cells treated with THC and WIN at a 50 µM final concentration. CBD treatment increases cox activity significantly by does not alter the ICE-1 level. The vehicle treatment (50 μM) does not affect the activity of ICE-1 or COX. The results are expressed as % change compared to the vehicle-treated control, with mean ± SD. * *p* < 0.05, ** *p* < 0.01, *** p < 0.001, **** *p* ≤ 0.0001. *n* = 5. (asterisk significant compared to the control).

Treatment with exogenous phytocannabinoids (CBD and THC) and a synthetic cannabinoid (WIN) increased oxidative stress, as seen by the significant increase in the generation of reactive oxygen species (ROS), NADH, and H_2_O_2_ and a concomitant decrease in the glutathione content (Figure 4A,B, *n* = 5, *p* < 0.05). However, regarding the variable effect of CBD and THC observed on the markers of oxidative stress analyzed, THC significantly increased lipid peroxidation; however, we could not demonstrate a significant effect of CBD on lipid peroxidation. A significant increase in the nitrite content was observed with CBD, but the opposite effect was observed with THC (Figure 4A, *n* = 5, *p* < 0.05).

### 3.4. CBD, THC, and WIN Treatments Induced Inflammation in Canine 1771 B-Cell Lymphoma Cell Line

Treatment of lymphoma cells with CBD, THC, and WIN induced inflammatory form of programmed cell death, as seen by the significant change in the activity of the inflammatory markers ICE-1 and COX (*n* = 5, *p* < 0.05; Figure 4C,D).

### 3.5. CBD, THC, and WIN Treatments Induced Apoptosis in Canine 1771 B-Cell Lymphoma Cell Line

**Figure 5:**
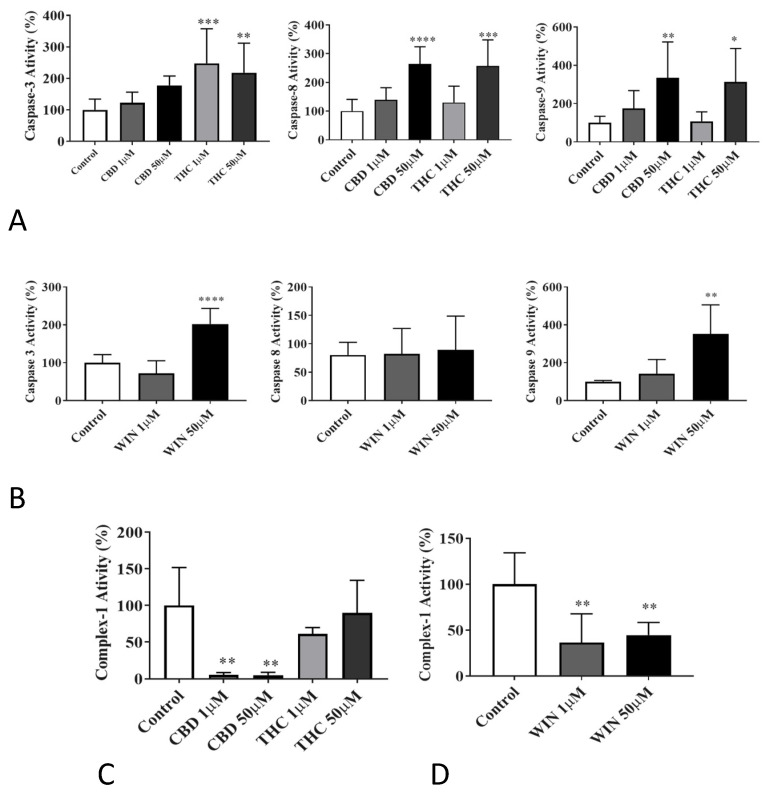
Effects of CBD, THC, and WIN on canine 1771 B-cell lymphoma cells. Caspase-3, caspase-8, and caspase-9 activities were measured spectrofluorometrically using AC-DEVD-AMC, Ac-VETD-AMC, Ac-LEHD-pNa, respectively, as the substrate. (**A**) CBD and THC increased all caspase activities in a dose-dependent manner. Significant dose-dependent effects on caspase-3 activity could not be demonstrated with CBD when compared to the control. (**B**) A significant increase in caspase-3 and caspase-9 activities was demonstrated in cells treated with WIN. The significant effect of WIN could not be demonstrated on caspase-8 activity. (**C**,**D**) Effects of CBD, THC, and WIN on canine 1771 B-cell lymphoma cells’ complex-I activity. The complex-I activity was measured spectrophotometrically using NADH as the substrate. CBD and WIN showed a significant decrease in complex-I activity compared to the control. The significant effect on complex-1 activity could not be demonstrated with THC. The results are expressed as % change compared to the control, with mean ± SD. * *p* < 0.05, ** *p* < 0.01, *** *p* < 0.001, **** *p* ≤ 0.0001. *n* = 5. (asterisk significant compared to the control).

Changes in the activity of various apoptotic markers indicated the apoptotic effect of the phytocannabinoids (CBD and THC) and the synthetic cannabinoid (WIN) against all lymphoma cell lines (Figure 5A,B). Treatment with THC caused a significant increase in all three of the caspases analyzed. Similarly, CBD and WIN increased the activities of caspase-3, caspase-8, and caspase-9 in a dose-dependent manner. The impact of CBD on caspase-3 and WIN on caspase-8 induction was appreciable, but it was not statistically significant.

### 3.6. Effects of CBD, THC, and WIN Cannabinoid Treatments on Mitochondrial Function

In canine B cells treated with CBD or WIN, complex-1 activity was significantly decreased when compared to the vehicle-treated control (*n* = 5, *p* < 0.001). In cells treated with THC, no significant effect on complex-1 activity could be demonstrated (Figure 5C,D and Table 2).

## 4. Discussion

It is well accepted that uncontrolled cellular growth, which may result from a defect in the cell cycle and apoptotic pathway, is one of the leading mechanisms for developing cancers, including lymphoma. Therefore, compounds that specifically induce apoptosis of cancer cells are likely to decrease tumor load and, consequently, be effective in cancer treatment. One of the most important and promising areas of current cannabinoid research is the ability of these natural products and endocannabinoids to modulate cell survival/death decisions, as described in several publications [6,20,41,42].

We recently studied endocannabinoid receptors and the effects of endocannabinoids on cell viability using canine and human NHL cell lines [21]. Our results demonstrated positive expression of cannabinoid receptors, CB_1_ and CB_2_, in both canine and human NHL cell lines, with a significantly higher expression in canine and human B-cell lymphoma compared to canine T-cell lymphoma. Also, we demonstrated decreased cell viability and apoptotic effect of endocannabinoids in canine and human NHL cell lines. The current study expanded our research from endogenous to exogenous cannabinoids. Among the exogenous cannabinoids studied, we focused on two phytocannabinoids, CBD and THC, and one synthetic cannabinoid, WIN. These selections were based on their well-documented anti-cancer effects across various human cancer models. Previous studies have highlighted the inhibitory effects of CBD and THC on cancer cell viability, along with their ability to trigger apoptotic mechanisms, leading to cell death in glioma, prostate, blood, breast, and other cancer cell lines [13,43,44,45,46]. Similarly, the synthetic cannabinoid WIN has been extensively studied for its potential anti-cancer activity against multiple cancers, including glioma, breast, and prostate cancers and mantle cell lymphoma [47,48,49,50].

Our objectives in this study were threefold: (1) an investigation of the inhibitory effects of CBD, THC, and WIN on canine and human NHL cell viability; (2) a study of the IC50 of phytocannabinoids (CBD and THC) and a synthetic cannabinoid(WIN) in canine and human NHL cell lines; and (3) an analysis of the effects of phytocannabinoids and a synthetic cannabinoid on oxidative stress, inflammation, apoptosis, and markers of mitochondrial function.

Our cell viability assay results demonstrated a growth stimulatory effect at lower cannabinoid concentrations. However, and in a clear contrast, a significant reduction in cell viability was consistently seen at higher non-molar final concentrations (25–50 µM) of the phytocannabinoids CBD and THC. Specifically, CBD significantly reduced cell viability in both B-cell (canine 1771 and CLBL1, and human Ramos) and canine CL-1 T-cell lymphoma cell lines. On the other hand, THC and the synthetic cannabinoid agonist WIN decreased cell viability significantly in only B-cell lymphoma cell lines. The lower expression of CB_1_ and CB_2_ receptors in CL-1 canine T-cell lymphoma cells, as compared to B-cell lymphoma reported in our previous study [21], could be the reason for the non-responsive behavior of CL-1 when treated with THC and WIN, both of which are selective cannabinoid receptor agonists. The hormesis-like effect of the lower phytocannabinoid concentrations on NHL cell viability seen in our study could be an adaptive response phenomenon previously documented in young adult C57BL/6 mice treated with a synthetic CP55940 cannabinoid and in other phytochemicals studies [51,52,53,54]. If future in vivo studies confirm our biphasic effect, the implications are that for some phytocannabinoids, too low a dose may be harmful, complicating individual responses. This phenomenon has been reported for resveratrol, a plant phenolic compound that induces a hormetic response in multiple well-characterized human cancer cell lines, including breast, prostate, colon, lung, uterine, and leukemia cell lines [55].

A further implication is that the hormesis-like response to CBD and THC could result in conflicting results among different studies if different concentrations are used. It is, thus, important to note that our understanding of the hormetic response to CBD and THC is very limited and further studies are needed to confirm this pharmacological phenomenon, especially in vivo.

The CBD-induced cytotoxicity in CL-1 T-cell lymphoma can potentially be mediated through receptors other than CB_1_ and CB_2_, as CBD has been shown in other studies to activate the transient receptor potential vanilloid subtype 1 (TRPV1), G protein-coupled receptor GPR55, the 5-HT1a receptor, and the α3 and α1 glycine receptors [56]. Several lymphoma studies have also demonstrated similar inhibitory effects of exogenous cannabinoids on cancer cell viability/proliferation [50,57,58,59].

To study the mechanism underlying the cytotoxic effects of CBD, THC, and WIN, markers of oxidative stress, inflammation, apoptosis, and mitochondrial function were analyzed in the 1771 B-cell lymphoma cell line. Our biochemical analysis results revealed a significant increase in ROS, H2O2, NADH, nitrite, and lipid peroxidation, and a concomitant reduction in the GSH content in cells treated with CBD, THC, and WIN at a 50 µM final concentration. Various studies from other laboratories have also demonstrated an increase in oxidative stress with exogenous and endogenous cannabinoid treatments in multiple cancer types, including human leukemia cells, human glioblastoma, and human glioma [46,60,61].

In our study, CBD, THC, and WIN treatments also significantly increased cyclooxygenase (COX) and Interleukin-1β-converting enzyme (ICE-1) activities. It is known that endocannabinoids are metabolized by COX-2 into pro-apoptotic prostaglandin derivatives, and both COX and ICE-1 enzymes are known to initiate pro-inflammatory mechanisms, leading to apoptosis or cell death [62,63,64]. The overproduction of COX and ICE-1 in cancer cells treated with CBD, THC, and WIN implies the possibility of the induction of an inflammatory pathway involved in cannabinoid-induced decrease in lymphoma cell viability. A similar effect of CBD has been previously demonstrated in glioblastoma [65,66].

Several cancer studies have demonstrated cannabinoid-induced apoptosis and associated this effect with increased caspase activity [46,61,67,68]. Likewise, we also demonstrated an increase in the activities of the apoptosis initiators Caspase-8 and -9 and the executioner Caspase-3 in lymphoma cells treated with the phytocannabinoids THC and CBD or with the synthetic cannabinoid WIN.

The inhibitory effect of cannabinoids on the mitochondrial respiratory chain has been demonstrated in past studies [44]. Similarly, compared to the vehicle-treated control cells, we found a significant reduction in complex-1 activity in 1771 B-cell lymphoma treated with WIN and THC when using concentrations as low as one micromole. However, we could not demonstrate a significant inhibitory effect of CBD on complex-1 activity. This differential effect of CBD could be attributed to the difference between these compounds triggering different anti-cancer signaling pathways. Further studies are needed to confirm this hypothesis.

In this study, we also analyzed the cytotoxic effects of phytocannabinoids (CBD and THC) and a synthetic cannabinoid (WIN) in lymphomas by calculating the IC50 value for each cell line. We found CBD to be the more potent phytocannabinoid (with a lower IC50) than THC. Overall, lymphomas with a higher expression of cannabinoid receptors responded more to the cannabinoid treatment compared to the ones with a lower expression (i.e., Ramos > CLBL-1 > 1771 > CL-1). However, the abovementioned anti-cancer effects can also be induced via mechanisms independent of CB1 and CB2 receptors.

## 5. Conclusions

Our study demonstrated a significant moderate inhibitory effect of CBD, THC, and WIN on canine and human NHL cell viability. Among the exogenous cannabinoids, the phytocannabinoid CBD was the most potent cannabinoid in 1771, Ramos, and CL-1, and the synthetic cannabinoid WIN was the most potent in the CLBL-1 cell line. Contrasting the inhibitory effect of CBD in B-cell versus T-cell lymphomas, we could not show a significant cytotoxic inhibitory effect of THC and WIN in the canine CL-1 T-cell lymphoma cell line. We surmised that the lack of a significant inhibitory effect may be due to the lower level of cannabinoid receptor expression in CL-1 T-cell cancer cells compared to B-cell lymphoma cell lines, as observed in our previous study [21].

Our results also revealed that CBD, THC, and WIN decreased lymphoma cell viability because they increased oxidative stress, leading to downstream apoptosis. Finally, our IC50 results could be lower than our findings due to serum binding. Furthermore, the results of our in vitro studies may not generalize to in vivo situations as many factors, including protein binding, could preclude direct extrapolation. In humans, THC may reach concentrations of approximately 1.4 µM in heavy users [69], and CBD may reach 2.5 µM [70] when administered orally therapeutically. Our study failed to demonstrate an inhibitory effect at these lower concentrations; the proliferative effects demonstrated in several cell lines with both CBD and THC may be problematic if these effects translate to in vivo responses. However, extrapolation of our in vitro results to in vivo situations would need to consider many other factors, including protein binding. This could preclude direct extrapolation. 

## Figures and Tables

**Table 1:** IC50 of phytocannabinoids and a synthetic cannabinoid in NHL cell lines.

Cell Line	Cannabinoid	IC50	SE *	R^2 †^
Canine B-cell lymphoma 1771	CBD	14 µM	2	0.9
	THC	41 µM	9.6	0.9
	WIN	NA	NA	NA
Canine B-cell lymphoma CLBL-1	CBD	18 µM	6.1	0.6
	THC	34 µM	2.2	0.9
	WIN	5.6 µM	0.6	0.9
Canine T-cell lymphoma CL-1	CBD	39 µM	3.2	0.8
	THC	NA	NA	NA
	WIN	NA	NA	NA
Human B-cell lymphoma Ramos	CBD	8.6 µM	1.3	0.9
	THC	21 µM	2.6	0.8
	WIN	14 µM	1.7	0.9

* SE: standard error, ^†^ R^2^: goodness of fit, NA: not applicable.

**Table 2:** Summary of biochemical analysis results.

Cellular Response to Cytotoxicity		Lymphoma Cells’ Response to Cannabinoid-Induced Cytotoxicity		
	Overall Effect	CBD	THC	WIN
Markers of Oxidative Stress				
ROS	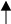	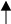	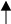	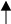
GSH	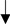	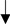	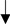	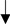
NADH	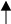	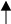	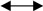	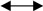
H_2_O_2_	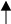	ND	ND	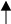
Nitrite	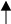	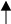	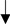	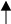
Lipid peroxidation (LP)	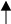	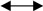	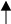	ND
Markers of Inflammation				
ICE-1	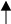	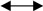	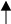	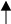
COX	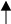	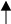	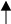	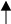
Markers of Apoptosis				
Caspase-3	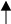	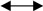	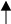	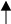
Caspase-8	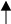	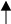	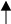	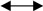
Caspase-9	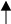	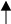	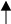	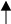
Markers of Mitochondrial function				
Complex-1	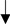	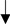	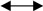	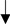

[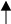 = Increased, 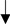 = Decreased, 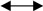 = No significant effect, ND = Not determined].

## Data Availability

The data that support the findings of this study are available from the corresponding author upon request. The data are not publicly available due to privacy or ethical restrictions.

## Supplementary Materials

The following supporting information can be downloaded at https://www.mdpi.com/article/10.3390/biom14040495/s1, Figure S1: Calculation of IC50 for AEA, 2AG, CBD, THC, and WIN using MTT—cell viability percent inhibition dose–response curves expressed as log 10 of concentration (in nM) vs. percent inhibition of 1771 cell viability; Figure S2: Calculation of IC50 for AEA, 2AG, CBD, THC, and WIN using MTT—cell viability percent inhibition dose–response curves expressed as log 10 of concentration (in nM) vs. percent inhibition of CLBL1 cell viability; Figure S3: Calculation of IC50 for AEA, 2AG, CBD, THC, and WIN using MTT—cell viability percent inhibition dose– response curves expressed as log 10 of concentration (in nM) vs. percent inhibition of Ramos cell viability; Figure S4: Calculation of IC50 for AEA, 2AG, CBD, THC, and WIN using MTT—cell viability percent inhibition dose–response curves expressed as log 10 of concentration (in nM) vs. percent inhibition of CL1 cell viability; Figure S5: Graphic representation of IC50 values of endocannabinoids (AEA and 2AG), phytocannabinoids (CBD and THC), and a synthetic cannabinoid (WIN) in NHL cell lines.
