# Supplementary material for: Effects of Cannabidiol, ∆9-Tetrahydrocannabinol, and WIN 55-212-22 on the Viability of Canine and Human Non-Hodgkin Lymphoma Cell Lines
Source: Biomolecules. 2024 Apr 19;14(4):495. doi: 10.3390/biom14040495 (PMC11047936; doi:10.3390/biom14040495)
Supplement: Supplementary file 1 [file biomolecules-14-00495-s001.zip › biomolecules-2939176-supplementary/Supplementary Material.pdf]

Supplementary Figures

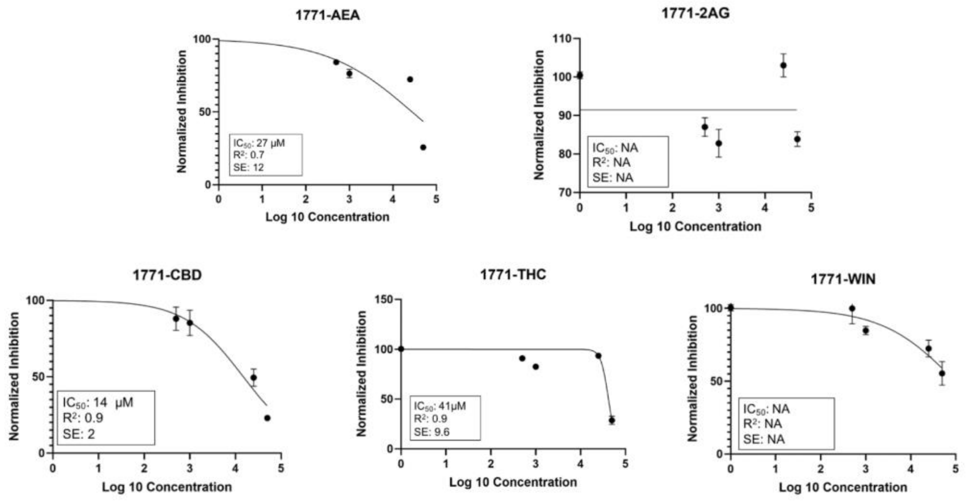

Supplementary Figure S1. Calculation of IC50 for AEA,2AG, CBD, THC and WIN using MTT-cell viability percent inhibition-dose response curves expressed as log 10 of concentration in (nM) vs percent inhibition of 1771 cell viability.

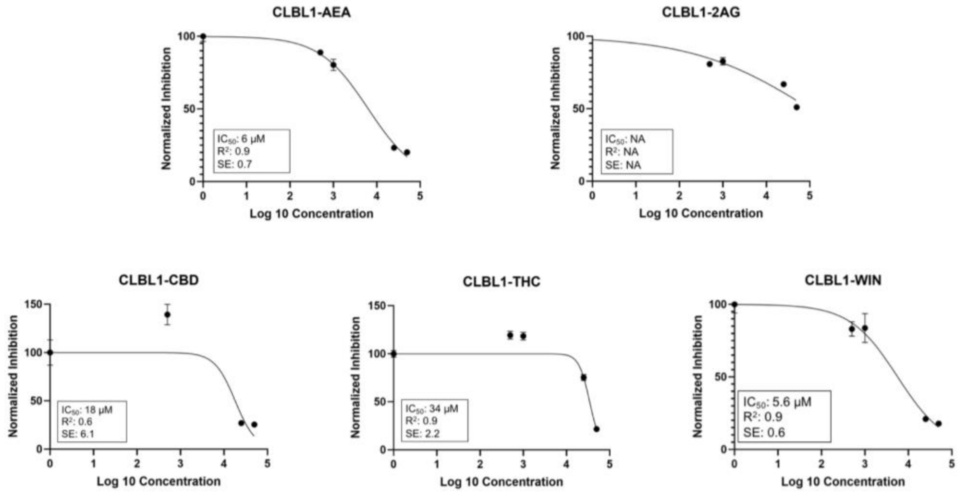

Supplementary Figure S2. Calculation of IC50 for AEA,2AG, CBD, THC and WIN using MTT- cell viability percent inhibition-dose response curves expressed as log 10 of concentration in (nM) vs percent inhibition of CLBL1 cell viability.

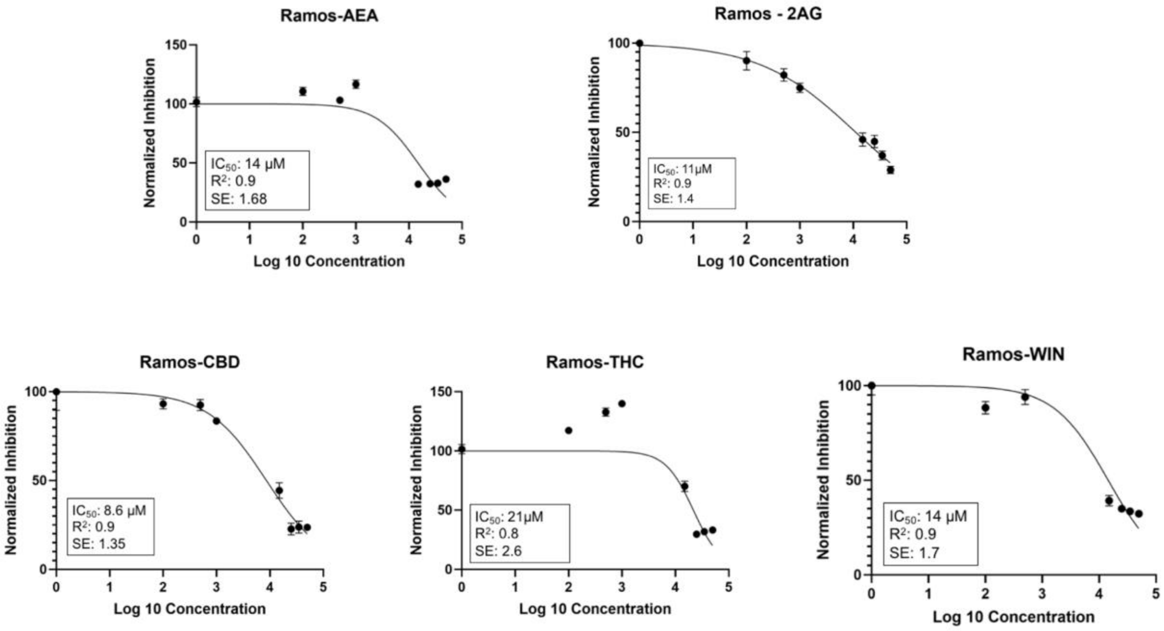

Supplementary Figure S3. Calculation of IC50 for AEA,2AG, CBD, THC and WIN using MTT- cell viability percent inhibition-dose response curves expressed as log 10 of concentration in (nM) vs percent inhibition of Ramos cell viability.

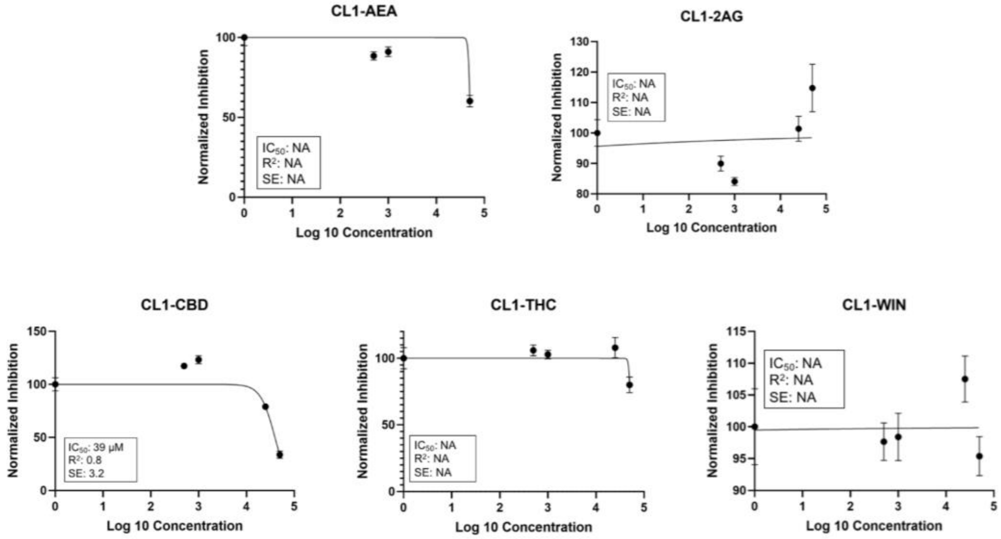

Supplementary Figure S4. Calculation of IC50 for AEA,2AG, CBD, THC and WIN using MTT- cell viability percent inhibition-dose response curves expressed as log 10 of concentration in (nM) vs percent inhibition of CL1 cell viability.

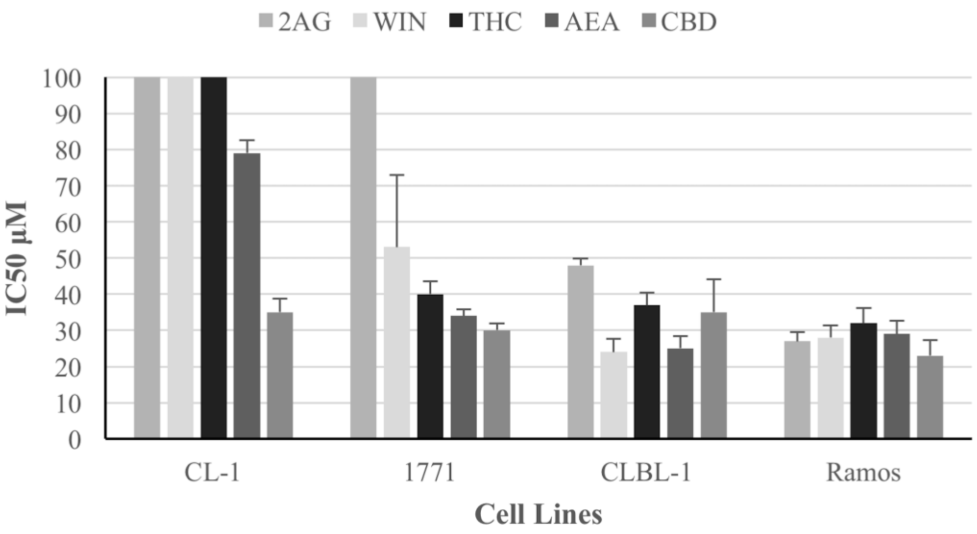

Supplementary Figure S5. Graphic representation of IC50 values of endocannabinoids (AEA, 2AG), phytocannabinoids (CBD, THC), and a synthetic cannabinoid (WIN) in NHL cell lines.
